# RNAi in Insect Physiology: Unlocking Mechanisms and Pioneering Sustainable Pest Control

**DOI:** 10.3390/insects17030333

**Published:** 2026-03-19

**Authors:** Jisheng Liu, Luc Swevers

**Affiliations:** 1 School of Life Sciences, Guangzhou University, Guangzhou 510006, China; 2 Institute of Biosciences and Applications, National Centre for Scientific Research Demokritos, 15341 Athens, Greece

## 1. Unveiling the RNAi Landscape: From Core Mechanisms to Applied Frontiers

The advent of RNA interference (RNAi) in 1998, marked by the discovery of gene silencing triggered by double-stranded RNA (dsRNA) in nematodes, opened a transformative chapter in molecular biology [1]. Today, RNAi has matured into an indispensable tool for elucidating gene function, while its potential as a targeted, eco-friendly pest control strategy is reshaping entomological research and agricultural practices [2]. This Special Issue of Insects, dedicated to “RNAi in Insect Physiology”, stands as a testament to the dynamic progress of RNAi research, bridging fundamental insights into insect physiology with groundbreaking applications in pest management.

At its core, RNAi encompasses a sophisticated network of gene silencing pathways, driven by small RNAs, including small interfering RNAs (siRNAs), microRNAs (miRNAs), and PIWI-interacting RNAs (piRNAs). These pathways collectively fine-tune gene expression in eukaryotic cells, offering precise molecular control over biological processes [3]. What makes RNAi particularly compelling in entomology is its unique mechanism of sequence-specific gene silencing, a feature that not only revolutionizes gene functional studies but also addresses the urgent need for sustainable pest control as traditional chemical pesticides face growing challenges of resistance and environmental harm [4].

This Special Issue was conceived to capture the full spectrum of RNAi research in insects with a dual focus: advancing our understanding of gene function in insect physiology and accelerating the translation of RNAi into practical pest control solutions. The 14 contributions featured here collectively paint a vivid picture of RNAi’s power, unlocking the molecular underpinnings of insect development, reproduction, and stress adaptation, while simultaneously demonstrating its viability as a next-generation pest management tool.

## 2. Highlights of the Special Issue: Bridging Mechanism and Impact

This Special Issue brings together 12 cutting-edge research articles and 2 insightful review articles, collectively showcasing the multifaceted power of RNAi in unraveling insect physiology and driving innovative pest control strategies.

On the front of gene function and pest control target discovery, key studies make significant strides. Research on the tomato leafminer *Tuta absoluta* identifies Akt, a serine/threonine kinase, as a central regulator of molting, hormones, and reproduction, highlighting its potential as an RNAi pest control target (Contribution 1). Complementing this, another work on the same pest characterizes 11 chitinase genes critical for larva–pupa–adult transitions, establishing them as promising targets for RNAi-based insecticidal development (Contribution 2). For the Colorado potato beetle *Leptinotarsa decemlineata*, silencing the *Miniature* gene effectively disrupts elytral and hindwing structures, offering a novel RNAi target to curb its dispersal and resistance (Contribution 3).

The issue also advances RNAi application in vector and pest management. For *Aedes albopictus*, a key Dengue fever vector, silencing genes in the 20E synthetic pathway demonstrate effective mosquito control, while assessing the environmental impacts of recombinant RNAi microalgae delivery (Contribution 4). In *Culex* mosquitoes, dsRNA-mediated knockdown of opsins eliminates UV-light-mediated attraction, enhancing trap efficacy (Contribution 5). Additionally, a study on *Anopheles sinensis*, a major malaria vector, reveals the involvement of carboxylesterase genes in pyrethroid resistance through transcriptome and functional analyses, providing a molecular basis for combating insecticide resistance in disease vectors (Contribution 6). Research on the soybean aphid *Aphis glycines* links high-temperature-induced heat shock protein 90 expression to insecticide sensitivity (Contribution 7), and studies on the brown planthopper *Nilaparvata lugens* conduct risk assessments for RNAi-based pesticides, ensuring safety for both target pests and non-target organisms (Contribution 8).

Beyond control, the issue deepens understanding of insect physiological adaptation and molecular mechanisms. Work on the wheat pest *Sitodiplosis mosellana* (Diptera: Cecidomyiidae) characterizes small heat shock protein genes involved in diapause, shedding light on stress adaptation (Contribution 9). For the brown planthopper, the study clarifies how trehalase regulates reproductive pathways and triglyceride metabolism, explaining its high reproductive capacity (Contribution 10). In the silkworm *Bombyx mori*, research reveals how *Escherichia coli* activates *BmToll9-1* to regulate growth, advancing insights into insect immune regulation (Contribution 11), a study that has completed the systematic investigation of the silkworm *BmToll9s* gene [5,6,7,8]. Moreover, a breakthrough study in *Trichogramma* wasps reports a stage- and species-specific RNAi system, overcoming technical barriers for molecular studies in these minute parasitoids (Contribution 12).

The two review articles provide critical context and forward-looking perspectives. One focuses on odorant binding proteins in *Tribolium castaneum*, exploring their functional diversity beyond olfaction and emerging applications in pest control (Contribution 13). The other offers a comprehensive overview of RNAi, contact unmodified antisense DNA biotechnology (CUADb), and clustered regularly interspaced short palindromic repeats (CRISPR) and CRISPR-associated proteins (CRISPR/Cas) as innovative silencing technologies, outlining their synergies and potential to transform pest management from discovery to practice (Contribution 14).

Together, these contributions bridge fundamental RNAi mechanisms with tangible impact, advancing both basic entomological research and practical pest control solutions.

## 3. Looking Ahead: The Promise of RNAi in Entomology

As we reflect on the contributions in this Special Issue, it is clear that RNAi is not just a research tool; it is a catalyst for innovation in entomology [9]. The studies presented here have expanded our understanding of insect physiology, identified novel targets for pest control, and developed new methodologies to harness RNAi’s potential. However, the journey is far from complete.

Looking ahead, several critical directions demand further exploration. First, improving the efficiency and specificity of RNAi delivery remains a key challenge, particularly for insects with diverse physiological barriers [2]. Advances in nanotechnology, microalgae-based delivery, and other innovative platforms will be crucial to overcoming these hurdles [10,11]. Second, understanding the off-target effects and environmental impacts of RNAi-based pest control strategies is essential to ensure their safe and sustainable deployment [12]. Third, integrating RNAi with other emerging technologies, such as CRISPR/Cas, synthetic biology, and ecological modeling, will unlock new synergies, enabling more comprehensive and resilient pest management solutions [13]. Fourth, dsRNA, as a pathogen-associated molecular pattern molecule, has the potential to interact with other immune defense mechanisms, which may complicate its use in pest control [14].

Moreover, as climate change and global trade continue to alter pest distributions and dynamics, the need for adaptive, environmentally friendly pest control strategies has never been greater. RNAi, with its specificity, scalability, and potential for integration into integrated pest management systems, is uniquely positioned to meet this challenge, embodying the core vision of One Health by safeguarding the health of agricultural ecosystems, thereby bridging crop protection with broader environmental and public health priorities [15].

## 4. Closing Remarks

This Special Issue on “RNAi in Insect Physiology” captures a pivotal moment in the evolution of RNAi research, a moment where fundamental insights converge with practical applications to address some of the most pressing challenges in entomology. The work presented here not only advances our knowledge of insect molecular biology but also paves the way for a new era of sustainable pest management, one that prioritizes ecological balance and human well-being.

We hope that this collection will inspire further research, foster collaboration across disciplines, and accelerate the translation of RNAi from the laboratory to the field. As the field of entomology continues to evolve, RNAi will undoubtedly remain at the forefront of innovation, driving discoveries that protect crops, safeguard public health, and preserve ecosystems.

We thank all our readers for engaging with this Special Issue and invite the scientific community to continue pushing the boundaries of RNAi research. Together, we can harness the power of this remarkable technology to build a more sustainable future for agriculture and the environment.

## Data Availability

No datasets were generated or analyzed.
